# Epidemiology, clinical aspects, and management of pediatric drowning

**DOI:** 10.1186/s13052-023-01464-1

**Published:** 2023-06-14

**Authors:** Francesco Pellegrino, Irene Raffaldi, Roberta Rossi, Barbara De Vito, Manuela Pagano, Davide Garelli, Claudia Bondone

**Affiliations:** 1 Department of Pediatric and Public Health Sciences, Postgraduate School of Pediatrics, Regina Margherita Children Hospital, University of Turin, Turin, Italy; 2 Department of Pediatric Emergency, Regina Margherita Children’s Hospital - A.O.U. Città della Salute e della Scienza di Torino, Turin, Italy

**Keywords:** Drowning, Injury prevention, Emergency medicine

## Abstract

Drowning is the third leading cause of injury death in the pediatric population worldwide, with incidence peaking among those aged 1–4 years and again in adolescence.

The purpose of this commentary is to review the basic pathophysiology of drowninginjury and factors that affect the outcome, such as submersion and hypothermia. We also discuss principles of prehospital and in-hospital management, comprising resuscitation and stabilization, administration of oxygen and intravenous liquids, and central reheating.

Even though the mortality rate has decreased in recent years, further investments and safety measures are needed to prevent child drowning deaths.

**Supplementary Information:**

The online version contains supplementary material available at 10.1186/s13052-023-01464-1.

## Background

Drowning is defined as “the process resulting in primary respiratory impairment from submersion/immersion in a liquid medium,” independent of whether it is fatal. This definition was developed after extensive debate by the first World Congress on Drowning (WCOD) held in Amsterdam in 2002 [1]. Before that time, authors used to differentiate fatal and nonfatal events, as well as “dry” and “wet” drowning, depending on the presence of fluid in the lungs or “active” and “passive” drowning, depending on whether the drowning was witnessed [1]. Currently, any water-distress incident without evidence of respiratory impairment (e.g., without aspiration) should be considered a water rescue and not a drowning.

Drowning is estimated to be responsible for 372,000 deaths annually worldwide, excluding those in natural disasters, making it the third most common accidental mortal injury and a major public health issue [2]. Moreover, there are geographical differences. Drowning rates in low- and middle-income countries are more than 90% of the total, up to 3.4-times higher than the rates in high-income countries [3]. The circumstances and rates of drowning also vary by age and gender. In the pediatric setting, the highest drowning rate has been observed in children between the first and fourth years of life, with a second peak in adolescents aged between 15 and 19 years [2]. Males are at greater risk of drowning than females up to 1 year of age, and among adolescents the gap increases 10 times for boys [4]. In infants, who are less mobile and entirely dependent on caregivers, drowning is often the result of inadequate supervision by adults. In early childhood, it is caused by the natural curiosity and lack of perception of danger, especially in the absence of barriers and close supervision. In adolescence, the main danger is represented by risky behaviors, such as diving, alcohol, or drug abuse, such as in adults.

The role of alcohol in drowning has been investigated extensively. A blood alcohol level of 0.10 g/100 ml may increase the risk of death associated with aquatic recreational activities approximately 10 times compared to individuals who are sober and involved in the same activities. The percentage of drowning deaths attributed to alcohol consumption has been estimated to be between 10% and 30% [2, 5].

Beyond the demographic characteristics and socio-economic status, underlying medical conditions have been investigated as a risk for drowning, particularly neurological conditions. Drowning is the most common cause of death from unintentional injury for people with epilepsy, intellectual disabilities, and autism spectrum disorders. Epileptic children are at greater risk of drowning, whether in bathtubs or swimming pools, with a rate 7.5 to 10-times higher than in children without seizures [6]. Children affected by autism spectrum disorder present with an increased risk of drowning [7, 8], and other pathologies found in drowning victims are silent or unrecognized heart disease, especially channelopathies, such as polymorphic catecholaminergic tachycardia and long QT syndrome, of which drowning is often the first dramatic consequence. Tester et al. reported 35 cases of drowning, 28.6% of whom presented with an unrecognized channelopathy [9].

The risk factors of intentional drowning deaths are under-explored. A recent Australian review identified adolescence, female gender, and psychoactive substance use as risk factors for suicidal drowning deaths, pointing out the lack of investigation about this topic [10]. The aim of this commentary is to highlight demographic and the socio-economic characteristics of specific age groups, underlying medical risk factors, and potentially dangerous circumstances that might allow more specific approaches to drowning prevention.

## Physiopathology

During drowning, the individual holds their breath consciously until the internal boost to inspire becomes irrepressible and they inhale water. The fluid reaches the airways, stimulating the cough reflex and laryngospasm. At this point, water causes surfactant loss, leading to consumption of the alveolar-capillary membrane, with an increase in its permeability and subsequent generalized pulmonary edema. Pulmonary edema impairs gas exchange in the lungs, resulting in hypercapnia, acidosis, and hypoxemia. The progressive reduction in the arterial partial pressure of oxygen (pO2) interrupts the laryngospasm and an additional amount of water is inhaled. If the person is not rescued, aspiration of water continues and hypoxemia leads to cerebral hypoxia, which rapidly causes loss of consciousness and apnea. Hypoxia and acidosis contribute to cardiac dysfunction, with a risk of dysrhythmias usually characterized by the sequence of tachycardia, bradycardia, pulseless electrical activity, and asystole [11, 12]. The whole mechanism, from water distress to cardiac arrest, takes a few minutes; though, in rare situations, such as rapid hypothermia, it can go on for more than an hour [13].

The cold receptors response includes gasping, hyperventilation, increased cardiac output, peripheral vasoconstriction, and hypertension. These responses can increase the metabolic rate, which decreases breath-hold time, which is generally 60–90 s at a comfortable air temperature [14] and is reduced to just a few seconds in water colder than 15 °C. However, hypothermia can provide a protective mechanism during drowning because it reduces the consumption of oxygen in the brain, delaying cellular anoxia and adenosine triphosphate (ATP) depletion. Hypothermia reduces the cerebral oxygen consumption by approximately 5% for each 1 °C reduction in temperature within the range of 37 °C to 20 °C [15].

## Management

### Prehospital management

The timing of the deterioration of drowned patients makes recognition and early intervention by prehospital care providers necessary for positive outcomes. The first challenge is to recognize a person in difficulty in the water and to activate the lifeguard or send someone to call for help. In rescuing a drowned person, the safety of the rescuers is fundamental. If possible, they must act without entering the water, throwing any floating object at the victim or alternatively reaching them by boat. The mantra should be: “Reach, Throw, Row, Don’t Go” [13]. The rescuer will enter the water only if there are sufficiently safe environmental conditions, the exit point from the water is too far away, or the patient is unconscious or unable to grasp the object.

Once on land, the person who has drowned should be placed in a supine position with their trunk and head at the same level, and the rescuer should be looking for vital signs and proceed to cardiopulmonary resuscitation (CPR) if necessary. Basic life support (BLS) should be started early because it is a crucial factor for survival. It is essential to follow the ABC model, with attention to airway, breathing, and circulation, in that order [16].

As the primary goal in a drowned patient is the rapid resolution of hypoxemia, the sequence of compression-ventilation allows a better neurological outcome than compressions alone. In cases of drowning, the European Resuscitation Council (ERC) recommends five initial rescue breaths because air can have difficulty reaching the airways due to the presence of water. Chest compressions alone are not recommended in persons who have drowned [17].

The most frequent complication during a resuscitation is the regurgitation of stomach contents, which occurs in more than 65% of persons who require rescue breathing alone and in 86% of those who require complete CPR. Active efforts to expel water from the airway (e.g., by Heimlich maneuver) are not indicated due to the delay in starting compressions and ventilation can greatly increase the risk of vomiting, with a significant increase in mortality [18].

Injuries to the cervical spine occur in less than 0.5% of drowned people, and cervical spine immobilization is indicated only if a traumatic head or neck injury history is confirmed or suspected (e.g., accidents involving diving, waterskiing, surfing, or watercraft) or focal neurological deficits occur [19]. Furthermore, as the treatment of hypothermia is one of the main predictors of favorable outcome in a drowned child, early treatment will be especially important, even in an out-of-hospital setting. The patient must be dried and covered with dry clothes or sheets, avoiding hindering the resuscitation maneuvers. Drying the patient is useful also in the case of defibrillator use [16].

Therefore, once the patient has been stabilized, if conscious, the collection of an accurate medical history is essential for complete patient management.

### Hospital management

Each child or adolescent rescued from drowning should be delivered to the hospital for evaluation and monitoring. Upon arrival to the Emergency Department (ED), clinicians should evaluate and treat different conditions.

#### Respiratory support

As already pointed out, the primary objective in the drowning patient is the correction of hypoxia;

thus, the priority is providing adequate oxygenation and ventilation. In the case of a conscious and eupneic patient, the administration of low-flow oxygen may be sufficient. However, in the case of respiratory distress, it is necessary to follow more advanced therapeutic strategies, such as the initiation of high-flow oxygen therapy or non-invasive positive pressure ventilation. In the unconscious patient, it is necessary to proceed with intubation, possibly via a cuffed tube, to initiate invasive ventilation in the Intensive Care Unit (ICU).

#### Fluid administration

In most children who have been rescued from drowning, the circulation is adequate after oxygenation and restoration of normal body temperature. In the presence of signs of shock, administration of intravenous saline, in the form of 10 ml/kg boluses, is indicated. Peripheral venous access is the preferred route for drug administration in the prehospital setting. Intraosseous access is an alternative route. Continued monitoring for any signs of worsening or non-response to fluid administration is necessary. Early cardiac dysfunction can occur in patients with cardiogenic shock. If volume replacement fails to restore hemodynamic adequacy, echocardiography can help in the use of inotropic agents, vasopressors, or both [20].

#### Correction of hypothermia

As already mentioned, it is essential to ensure adequate “rewarming” in the hypothermic patient (defined as rectal temperature < 35 °C), as it is directly related to a better outcome. Hypothermia also reduces the effectiveness of defibrillation and emergency medications. According to the ERC guidelines, severe hypothermia modifies the Pediatric Advanced Life Support (PALS) algorithm by limiting the use of drugs and defibrillation:

- As long as the core temperature does not exceed 30 °C, the defibrillation shock limit is 3 and inotropes or antiarrhythmics are not recommended because they are less effective;
- Body temperature between 30 and 35 °C, the interval for emergency drug administration must be doubled (every 6–10 min).

Methods of heating:

1. Passive.
  - Removing wet dresses.
  - Covering with warming blanket.
  - Increasing environmental temperature.
2. Active (in case of internal temperature < 30 °C).
  - Parenteral administration of warm fluid (at 38–40 °C).
  - Inhalation of gases at 42 °C.
  - Gastric and urethral washing with saline solution at 42 °C.

The goal is to increase the body temperature by 0.5 °C/h until the target of 35 °C is reached.

In case of severe hypothermia (core temperature < 30 °C) and concomitant cardiac arrest, the use of extracorporeal membrane oxygenation (ECMO) is indicated in the shortest time possible to restore the body temperature [17].

Resuscitation maneuvers must be continued until the body temperature is at least 32 °C, whereas in non-hypothermic patients, they may be stopped after 20 min of CPR.

#### Correction of metabolic and electrolytic imbalances

Acidosis in the drowned patient is corrected by restoring normal oxygenation and ventilation by re-expanding the circulating volume and using inotropic agents. The routine use of sodium baking soda is not recommended.

It may be necessary to correct any electrolytic disorder, such as hypoglycemia, hyperkalemia, or hypophosphatemia because they can impair renal function. The use of corticosteroids is not indicated because they are ineffective and capable of interfering with the healing process, as well as surfactant use, as there is no good evidence that its use improves outcomes [13].

The routine use of antibiotics is also not recommended, except in cases of known infection or drowning in contaminated water. In the unconscious patient, the placement of a nasogastric tube and bladder catheter is indicated [16].

Diagnostic tests:

1. Blood gas analysis to assess the presence of acidosis, hypoglycemia, electrolyte alterations;
2. Blood chemistry tests, including kidney function and coagulation;
3. Possible toxicological examination if the dynamics of the event are unclear, especially in the adolescent patient;
4. 12-lead electrocardiography (ECG);
5. Chest X-ray is not routinely recommended except for patients with respiratory distress - the eventual presence of an infectious or chemical pneumonia can be detected 24 h after the event;
6. Brain or cervical spine computed tomography is not routinely recommended, but only in the case of altered consciousness, suspected concomitant severe head, and/or spinal injury.

When the patient is hemodynamically stable, it is useful to investigate any underlying conditions, such as epilepsy, hypoglycemia, and unrecognized cardiac arrhythmias, as well as the circumstances in which the drowning occurred to seek out any contributing factors, such as alcohol abuse, drug use, or history of abuse.

## Prognosis

Drowning is a major cause of mortality and morbidity in children worldwide. Different outcome predictors at the scene of drowning and upon arrival at the hospital have been studied to improve prevention and care. A meta-analysis was performed by Quan et al. considering six scene predictors: age of victim, submersion period, salt versus fresh water, water temperature, CPR, and hospital arrival time. The most relevant independent predictor was submersion duration. Shorter submersion was linked to better outcomes. In particular, submersion for ≤ 6 min predicted better outcomes, whereas submersion for > 10 min was related to bad outcomes. The highest risk ratio was submersion of 15 to 25 min, with duration > 25 min being invariably fatal. The child’s age was not a predictor, such as the role of cold water in preserving neurological function [21]. The prognosis in children also depends on the timing of resuscitation maneuvers. A delay in the start of CPR or no return of spontaneous circulation within 30 min of CPR is associated with a worse prognosis [22].

No evidence has been found of the protective effect of colder water temperatures in drowning victims, and hypothermia does not improve the chances of survival [21]. Suominen et al. reported no significant differences in outcomes between children and adults despite the theoretical advantage of a higher surface area to body weight ratio. Therefore, survival and neurological outcome after drowning in adults and children is critically dependent on the duration of submersion, which remains the best prognostic factor [23].

An option for future studies focusing on the identification of prognostic factors related to both survival and neurological outcomes from drowning may include the Pediatric Risk of Mortality (PRISM) score. It is a scoring system with 14 physiological variables and 23 variable ranges that predicts the mortality risk of critical illness in children [24].

## Discharging

In children who have mild symptoms and a normal state of consciousness, discharge is recommended 8 h after starting observation in the ED [25, 26]. The prognosis is excellent in children who are conscious at presentation. This protocol is supported by a retrospective, single center, pilot study led by Shenoi et al. They investigated children aged 0–18 years who were admitted to the tertiary-care children’s hospital ED after a drowning event in order to identify a cohort of children at low risk of submersion-related injury who can be safely discharged from the ED after a period of observation. They derived and validated five items for a submersion risk score: normal mentation, normal respiratory rate, absence of dyspnea, absence of need for airway support (bag-valve mask ventilation, intubation, and noninvasive ventilation), absence of systolic hypotension. A score ≥ 4 at arrival would suggest safe discharge after 8 h. Application of this score, if confirmed by further validation studies, could help ED physicians make decisions on the timing of discharge [26].

## Prevention

The World Health Organization’s Global Report on Drowning and the National Water Safety Forum’s UK National Drowning Prevention Strategy have recently focused on drowning prevention strategies [2]. People drown in different settings; therefore, the prevention strategies should be broad. First, they can focus on education regarding potential risks associated with swimming for children with epilepsy or channelopathies and other disorders. Second, they can focus on the use of multiple layers of safety barriers, such as pool fencing, pool covers, and water entry alarms. Additional prevention methods should focus on the importance of adult surveillance, lifeguard’s role, and the development of strategies, such as swimming and survival skills training [27].

### Physical barriers

Most drowning events, especially when young children are involved, take place inland [2]. A national survey in Bangladesh showed that 80% of drowning among children under 5 years of age occurred within 20 m of the house, mainly in ponds, followed by ditches and water containers [28]. Another study conducted in the rural community of Kaniyambadi, India, highlighted that almost 90% of drowning deaths among children between 1 and 12 years of age happened in a pot or pond [29]. Most of the children of Caucasian origin drowned in residential pools, whereas those of African origin were more likely to die in public pools, often at motels [30].

Strategically placing barriers is a method for decreasing exposure and drowning risk, and effective approaches vary: covering water tanks, using doorway barriers and playpens, fencing swimming pools with four-sided, child-resistant fences and self-closing gates with safety latches, as well as legislating for the implementation of these policies [2]. Pool fencing is the most studied and effective drowning-prevention strategy for the young child. It should completely encircle the pool and isolate it from the house. Legislation should require isolation fencing with secure, self-latching gates for all pools − public, semi-public, and private [31].

The safety of pool alarms has been studied and, though they functioned properly, no report has demonstrated whether pool alarms prevent drowning and concluded that alarms “should not be relied on as a substitute for supervision or a barrier completely surrounding the pool” [32].

#### Life jackets

Life jackets prevent drowning deaths; the use of an approved life jacket decreases boat-related drowning morbidity and mortality by 50% [33]. Unfortunately, their use remains low. Factors associated with little use include older age, male gender, discomfort, cost, alcohol consumption, and the perception of greater swimming ability [34]. Legislation and educational campaigns are the most effective way to increase life jacket use, leading to 90–95% compliance among specific groups, such as children, people on personal watercraft, and those in water activities [35].

#### Lifeguards and CPR training

We already underlined the importance of the timing of resuscitation maneuvers on drowning outcomes. If CPR is performed immediately, victims of drowning have a better clinical outcome [36]. Considering the crucial role of effective CPR, Fernàndez-Méndez et al. studied the ability of 20 professional lifeguards to perform CPR, concluding that improving lifeguard training programs is necessary [37]. On the other hand, a survey reported that 20% of parents interviewed thought that the lifeguard was the main person responsible for supervising their child while in the water, leading to a false sense of security and a lack of parental supervision [38]. However, lifeguards must not take the place of caregiver supervision.

#### Adult supervision

Adequate supervision of young children is described as close, attentive, and constant supervision of young children when they are in or around any body of water, and it is an essential preventive strategy [39]. Appropriate supervision also includes examination of any unfamiliar environment, such as an unfenced pool or pond, and prevention measures, such as locked doors and close gates, especially for younger children. Unfortunately, parents and caregivers may have misperceptions about situations and dynamics that may lead to drowning, and they do not appropriately supervise children, who are often alone when they drown [40, 41].

Some parents promote “water watchers,” an encouraging program in which a designated adult is responsible for constant supervision without engaging in any distracting activities. Moreover, a promising program called “S.A.F.E.R. Near Water” (Supervise by Always being Focused on the children and able to Extend your arms and Reach them) has been developed and tested, showing that parents who completed it acquired better perceptions of the value of supervision and better judgment about children’s swimming skills and drowning risk [42].

#### Swimming lessons and water competency

Children aged 2–4 years can acquire the motor skills for swimming [43]. Brenner et al. found that the preschool-aged group had a reduction in the fatal drowning risk if they attended swimming lessons [44]. Swimming lessons should include parental training to improve parents’ understanding of their child’s actual swimming abilities and continued risk.

The international community focused on drowning prevention has started to expand the concept of water competency to include needed skills, knowledge, and behaviors [45]. In addition to basic swimming skills, water competency should include knowledge of local hazards in the aquatic environment, risk judgment, and self-assessment of abilities. In addition, swimming lessons are increasingly available for children with various disabilities, including autism or other health conditions.

## Conclusion

In summary, even if the mortality rate in children has decreased in recent years, childhood drowning is still a major burden in public health. Recognition of the demographic and socio-economic characteristics, clinical risk factors, and potentially critical circumstances may allow more specific approaches to drowning prevention. Prevention measures should focus on increasing public awareness of the risks of children swimming unsupervised. In addition, it is essential to ensure early resuscitation of drowning victims by promoting life support courses to facilitate positive outcomes.

## Electronic supplementary material

Below is the link to the electronic supplementary material.

Supplementary Material 1

## Data Availability

Data available on request due to privacy/ethical restrictions.

## List of Abbreviations

WCOD: World Congress on Drowning
GBD: Global Burden of Diseases
pO2: Partial pressure of oxygen
ATP: Adenosine triphosphate
CPR: Cardiopulmonary resuscitation
BLS: Basic life support
ED: Emergency Department
ICU: Intensive Care Unit
ERC: European Resuscitation Council
PALS: Pediatric Advanced Life Support
ECMO: Extracorporeal membrane oxygenation
ECG: Electrocardiography
PRISM: Pediatric Risk of Mortality
S.A.F.E.R.: Supervise by Always being Focused on the children and able to Extend your arms and Reach them
