# Supplementary material for: Epidemiology, clinical aspects, and management of pediatric drowning
Source: Ital J Pediatr. 2023 Jun 14;49:74. doi: 10.1186/s13052-023-01464-1 (PMC10268379; doi:10.1186/s13052-023-01464-1)
Supplement: Supplementary file 1 — Supplementary Material 1 [file 13052_2023_1464_MOESM1_ESM.docx]

Torino, April 02^nd^, 2023

To ­Editor and Reviewer of IJP

Dear Editor and Reviewer,

Thank you for your suggestions.

We restructured the manuscript following the journal’s guidelines for review and inserted a new “Background” paragraph in which we summarized the issue. Unfortunately, no differences in outcome between children and adults have been reported in the literature. We added a short paragraph about this with a new reference.
The aim of our review is not to add something new about this topic, but to outline the state of the art and focus attention on a relevant underestimated condition, extensively underlining the importance of pre-hospital management and prevention strategies. We dedicated a large part of the article to these topics. This is the main difference between our paper and others that have focused on hospital management and emergency protocols.

We also improved the quality of the English, submitting our manuscript to an editing service.
We hope you can accept the revised version of our paper.

Sincerely,

Francesco Pellegrino, MD

Claudia Bondone, MD
